# Caizhixuan hair tonic regulates both apoptosis and the PI3K/Akt pathway to treat androgenetic alopecia

**DOI:** 10.1371/journal.pone.0282427

**Published:** 2023-02-24

**Authors:** Tingting Fang, Ruofei Xu, Shaopeng Sun, Yineng He, Yi Yan, Hongyang Fu, Hongbin Luo, Yi Cao, Maocan Tao

**Affiliations:** 1 The First Clinical Medical College of Zhejiang Chinese Medical University, Hangzhou, Zhejiang, China; 2 Longyou County People’s Hospital, Longyou, Zhejiang, China; 3 The First Affiliated Hospital of Zhejiang Chinese Medical University, Hangzhou, Zhejiang, China; Foshan University, CHINA

## Abstract

**Purpose:**

Caizhixuan hair tonic (CZX) is a topical traditional Chinese medicine (TCM) preparation for the treatment of androgenetic alopecia (AGA). However, its active compounds and underlying mechanism for treating AGA are still unclear. The purpose of this study was to observe the effects of CZX on hair growth promotion in AGA mice and to explore the active components and mechanism.

**Methods:**

Testosterone propionate was administered subcutaneously to mice to establish an AGA mouse model. The therapeutic effects of CZX on AGA were evaluated by observing skin colour changes, hair growth time, and average hair length; calculating the hair growth score; and performing skin histopathological analysis. Following that, CZX chemical components were analysed by ultra-high-performance liquid chromatography-quadrupole-time-of-flight mass spectrometry (UPLC–Q–TOF/MS). Network pharmacology was used to predict the major effects and possible mechanisms of CZX for the treatment of AGA. Furthermore, RT-qPCR and Western blotting were performed to assess the expression of key genes and proteins involved in PI3K/Akt and apoptosis pathways in order to validate CZX’s predicted mechanism in AGA.

**Results:**

CZX promoted hair growth and improved the pathological morphology of hair follicles in the skin. In UPLC–Q–TOF/MS analysis, 69 components from CZX were isolated. Based on network pharmacology, CZX alleviated AGA by regulating PI3K/Akt and apoptosis pathways. According to RT-qPCR and Western blotting, CZX upregulated the expressions of PI3K, Akt, and Bcl-2, while downregulating that of Bax and caspase-3.

**Conclusions:**

CZX promotes hair growth to treat AGA by regulating the PI3K/Akt and apoptosis pathways.

## Introduction

Androgenetic alopecia (AGA) is the most common type of non-scar alopecia found in the clinic and is characterized by progressive hair follicle miniaturization and perifollicular microinflammation [1]. AGA occurs in 50% of adult women and 80% of adult men. Its incidence is increasing year by year and it is appearing in younger individuals [2]. Although AGA does not cause direct harm to the body, it may bring serious psychological pressure to patients, resulting in negative emotions such as inferiority, anxiety and depression and seriously affecting personal social activities and quality of life [3]. Therefore, treating hair loss is necessary to maintain the physical and mental health of patients. At present, the effective rates of finasteride and minoxidil, which have been approved by the Food and Drug Administration (FDA) for the treatment of AGA, are 48% and 35%, respectively [4]. However, wide application of these two drugs in the clinic is limited due to the side effects caused by long-term administration, such as sexual dysfunction, facial hairiness and scalp stimulation [5–7]. Therefore, it is crucial to actively seek new drugs that effectively promote hair growth.

In recent years, traditional Chinese medicine (TCM) has achieved good results with wide recognition for the treatment of AGA [8]. Caizhixuan hair tonic (CZX) is a topical preparation that is extracted from Chinese herbs with ethanol, and previous clinical practices have indicated that it has a therapeutic effect on AGA. CZX contains six Chinese herbs: *Polygonum multiflorum* Thunb. (Heshouwu), *Sesamum indicum* L. (Heizhima), *Astragalus membranaceus* (Fisch.) Bge. (Huangqi), *Platycladi cacumen* (Cebaiye), *Zingiber officinale* Rosc. (Shengjiang) and *Angelica sinensis* (Oliv.) Diels (Danggui). TCM is highly dependent on *P*. *multiflorum*, *A*. *sinensis* and *S*. *indicum* for the treatment of AGA [9,10]. Topical application of *P*. *multiflorum* significantly increased the hair follicle quantity and extended the hair follicle length in experimental animals [11]. *A*. *sinensis* can regenerate hair by inhibiting apoptosis in the regression period [12]. Zhang Y et al. found that volatile oil from *P*. *cacumen* could prolong the growth period of hair follicles in vitro, and its topical application could promote the growth of mouse hair in vivo [13]. Astragaloside IV, the active ingredient of *A*. *membranaceus*, inhibits apoptosis-regression catagen in hair follicles, leading to hair regrowth [14]. Clinical studies have shown that prescriptions containing herbs from Zingiberaceae have significant therapeutic effects on alopecia [15]. Nevertheless, the bioactive components and pharmacological mechanism of CZX are still unclear.

In this study, we used ultra-high-performance liquid chromatography-quadrupole-time-of-flight mass spectrometry (UPLC–Q–TOF/MS) to rapidly screen and systematically identify the chemical composition of CZX. The main components, pivotal targets and signalling pathways of CZX for the treatment of AGA were identified by network pharmacology and validated by in vivo experiments to thoroughly explain the relationship between CZX and AGA.

## Materials and methods

### Materials

CZX was supplied by Hangzhou Senkuo Biological Technology Co., Ltd. (WHBD273B, Hangzhou, China), and 2% minoxidil was supplied by Shanxi Zhendong Anxin Biopharmaceutical Co., Ltd. (H20020190, Shanxi, China). Testosterone propionate injection was purchased from Ningbo Second Hormone Factory (210204, Ningbo, China). Soybean oil was purchased from Shanghai Aladdin Biochemical Technology Co., Ltd. (S110245, Shanghai, China). The rabbit polyclonal anti-β-actin antibody was purchased from Dawen Biotechnology (DW130656, Hangzhou, China). Mouse monoclonal anti-Bcl-2 and anti-PI3K and rabbit monoclonal anti-Bax antibodies were purchased from Hangzhou HuaAn Biotechnology Co., Ltd. (EM1701-82, EM1701-62, ET1603-34, Hangzhou, China). Rabbit monoclonal anti-Akt and anti-caspase-3 antibodies were purchased from Cell Signaling Technology (4060S, 9662S, Danvers, MA, USA). The RNA Isolation Kit and cDNA Synthesis Kit were obtained from Wuhan Servicebio Technology Co., Ltd. (G3013, G3330, Wuhan, China).

### Preparation of the sample solution

CZX was diluted via mixing with 95% ethanol in a ratio of 1:4 (v/v) and then filtered through a 0.22 μm filter membrane; afterwards, the filtrate was used for UPLC–Q–TOF/MS analysis.

### Animal experiments

Male C57BL/6 mice (6 weeks of age) were obtained from Hangzhou Medical College (Certificate No.: SCXK(ZHE)2019-0002). Experiments on animals were carried out according to the regulations of Zhejiang Chinese Medical University’s Experimental Animal Ethics Committee (Approval No.: IACUC-20210322-05).

A week after adapting to feeding, mice were divided into four groups at random (n = 8): the normal group, model group, CZX group and minoxidil group. A 2 cm × 3 cm dorsal hair section of these mice was depilated with depilatory cream. Beginning the next day, 20 μL of 0.5% testosterone propionate injection (diluted with soybean oil) was injected subcutaneously into the shaved dorsum of each mouse once daily for 28 days, excluding the normal group. Half an hour after the application of testosterone, 100 μL of the corresponding drug solution was applied topically to each group of mice separately as follows: the minoxidil group was given 2% minoxidil, the CZX group was given CZX hair tonic, and the normal and model groups were given 95% ethanol solution as controls.

Daily observations were made of the colour of the skin in the depilated area of each group, as well as when the colour changed from pink to black and when the hair began to grow. From the 8th day after topical administration, hair regrowth was scored every other day to be able to dynamically observe the rate of hair growth. We scored hair growth according to the percentage of hair growth in the depilated area: 0 to 20% was scored as 1, 20% to 40% as 2, 40% to 60% as 3, 60% to 80% as 4 and 80% to 100% as 5 [16]. After 28 days of treatment, the mice were euthanized by carbon dioxide asphyxiation, and the skin of the dorsal experimental area was obtained for further experiments. Five hairs were randomly extracted from the depilated area of each mouse, and their lengths were measured with a Vernier calliper.

### Histological analyses

The excised dorsal skin of each mouse was immersed in 4% paraformaldehyde. After conventional ethanol gradient dehydration, the skin samples were embedded in paraffin and sectioned into 4 μm slices, including longitudinal and transverse patterns, and then stained with haematoxylin and eosin (HE). The longitudinal sections were used to observe the hair follicle morphology, while the transverse sections were used to count the hair follicles.

### UPLC–Q–TOF/MS analysis

Qualitative analysis of CZX was performed by UPLC (Waters, Milford, MA, USA). The chromatographic separation was performed on a Waters CORTECS UPLC T3 column (2.1 × 100 mm, 1.6 μm). The mobile phase was composed of 0.1% aqueous formic acid solution (A) and acetonitrile (B). The gradient elution conditions were as follows: 0 to 2 min, 5% B; 2 to 32 min, 5% to 100% B; 32 to 33 min, 100% B; 33.5 min, 5% B; 33.5 to 35 min, 5% B. The column temperature was set at 35°C, the flow rate was 0.3 mL/min, and the injection volume was 2 μL.

Electrospray ionization (ESI) sources were used to acquire mass spectrometry data from 50 m/z to 1200 m/z in both negative and positive ionization modes. The source parameters were as follows: ion source temperature of 120°C, desolvation temperature of 400 to 500°C, capillary voltage of 2.5 to 3.0 kV, sample cone voltage of 40 V, source offset voltage of 80 V, low collision energy of 6 V, high collision energy of 15 to 45 V, and desolvation gas flow rate of 800 to 1000 L/h.

### Network pharmacology analysis

#### Target prediction of the identified components

The compounds identified in CZX were subjected to network pharmacological analysis. Their 2D structures were obtained from the PubChem database (https://pubchem.ncbi.nlm.nih.gov/) and then imported into the SwissTargetPrediction database (http://www.swisstargetprediction.ch/) to predict the possible targets of the components with “*Homo sapiens*” selected as the species. Then, multiple targets were obtained for each chemical component to exert its pharmacological effects.

#### Collection of potential targets for AGA

The GeneCards (https://www.genecards.org/), OMIM (https://omim.org/), PharmGkb (https://www.pharmgkb.org/), and DrugBank (https://go.drugbank.com/) databases were used to search for genes related to AGA using the keyword “androgenetic alopecia”. The search results from the four databases were merged to remove duplicate targets to obtain the potential target genes for the treatment of AGA.

#### Construction of the protein–protein interaction (PPI) network

R language was used to obtain the intersection targets of the effective components of CZX and the potential targets of AGA and to draw a Wayne diagram. Based on the above analyses, the visual Component-target network was constructed using Cytoscape software. The intersection target genes were imported into the STRING database (https://cn.string-db.org/) to determine the relationship between the targets and obtain PPI network data with the species set as “*Homo sapiens*” and a minimum required interaction score of 0.4. Then, the obtained network was imported into Cytoscape 3.8.0, and the CytoNCA plug-in was used to calculate the score of each node. To identify core genes, betweenness centrality (BC), closeness centrality (CC), degree centrality (DC), eigenvector centrality (EC), local average connectivity-based method (LAC) and network centrality (NC) greater than the median were utilized as the screening criteria [17].

#### Gene Ontology (GO) and Kyoto Encyclopedia of Genes and Genomes (KEGG) pathway enrichment analyses

R language was used for GO and KEGG pathway enrichment analysis of intersection targets, and *P* < 0.05 was used as the screening condition. GO enrichment analysis included biological process (BP), cellular component (CC) and molecular function (MF).

#### Molecular docking

The PDB file of the core targets was downloaded from the RSCB PDB online platform (https://www.rcsb.org/), and the MOL2 file of the core active ingredients was downloaded from the PubChem database (https://pubchem.ncbi.nlm.nih.gov/). Molecular docking was performed with AutoDock Tools software (1.5.6, The Scripps Research Institute, La Jolla, CA, USA), and the minimum binding energy was recorded. The results were processed using PyMOL software (2.5.0, Schrodinger, Inc., New York, USA) and displayed in images.

### Western blot analysis

Total protein was extracted from the mouse skins, quantified by BCA protein assay and separated using SDS‒PAGE. Following separation, the proteins were transferred onto PVDF membranes and blocked with 5% skim milk for 1 h. Then, a primary antibody was infused onto the membranes overnight at 4°C. After 18 h, the secondary antibodies were added for 1 h of incubation followed by chemiluminescence visualization. Quantified protein levels were analysed using ImageJ software, and the relative expression of target protein was calculated by comparing the greyscale value of the target band with the β-actin band from the same sample.

### Quantitative real-time PCR analysis

Total RNA was extracted from skin according to the TRIzol method, and the concentrations and purities of the RNAs were measured using a Nanodrop 2000. cDNA was synthesized according to the instructions of the reverse transcription reaction kit. Real-time PCR was executed as described below: pre-denaturation at 95°C for 10 min, denaturation at 95°C for 15 s, annealing at 60°C for 30 s, and extension at 60°C for 30 s for a total of 40 cycles. The relative mRNA levels were assessed by the 2^-ΔΔ^Ct method with GAPDH as the reference. PCR primer sequences were synthesized by Wuhan Servicebio Technology Co., Ltd. and are shown in Table 1.

**Table 1:** Primer sequences used for PCR.

Gene	Upstream primer sequence (5′ to 3′)	Downstream primer sequence (5′ to 3′)
PI3K	AAACTCCGAGACACTGCTGATG	GCTGGTATTTGGACACTGGGTAG
Akt	CTTCCTCCTCAAGAACGATGGC	TGTCTTCATCAGCTGGCATTGT
Bcl-2	AGGATTGTGGCCTTCTTTGAGT	ACAGCCAGGAGAAATCAAACAGA
Bax	TTGCTACAGGGTTTCATCCAGG	GCAAAGTAGAAGAGGGCAACCA
Capspase-3	TGGAATGTCATCTCGCTCTGGT	GAAGAGTTTCGGCTTTCCAGTC
GAPDH	CCTCGTCCCGTAGACAAAATG	TGAGGTCAATGAAGGGGTCGT

### Statistical analysis

The statistical analysis was performed using GraphPad Prism 8.0.1. The data are expressed as the means ± SDs. In situations where the data conformed to a normal distribution, Student’s t test was used to compare differences between two groups, and one-way ANOVA was used for comparing differences between multiple groups. A *P* value less than 0.05 (*P* < 0.05) was considered statistically significant.

## Results

### CZX-mediated promotion of hair growth

**Fig 1:**
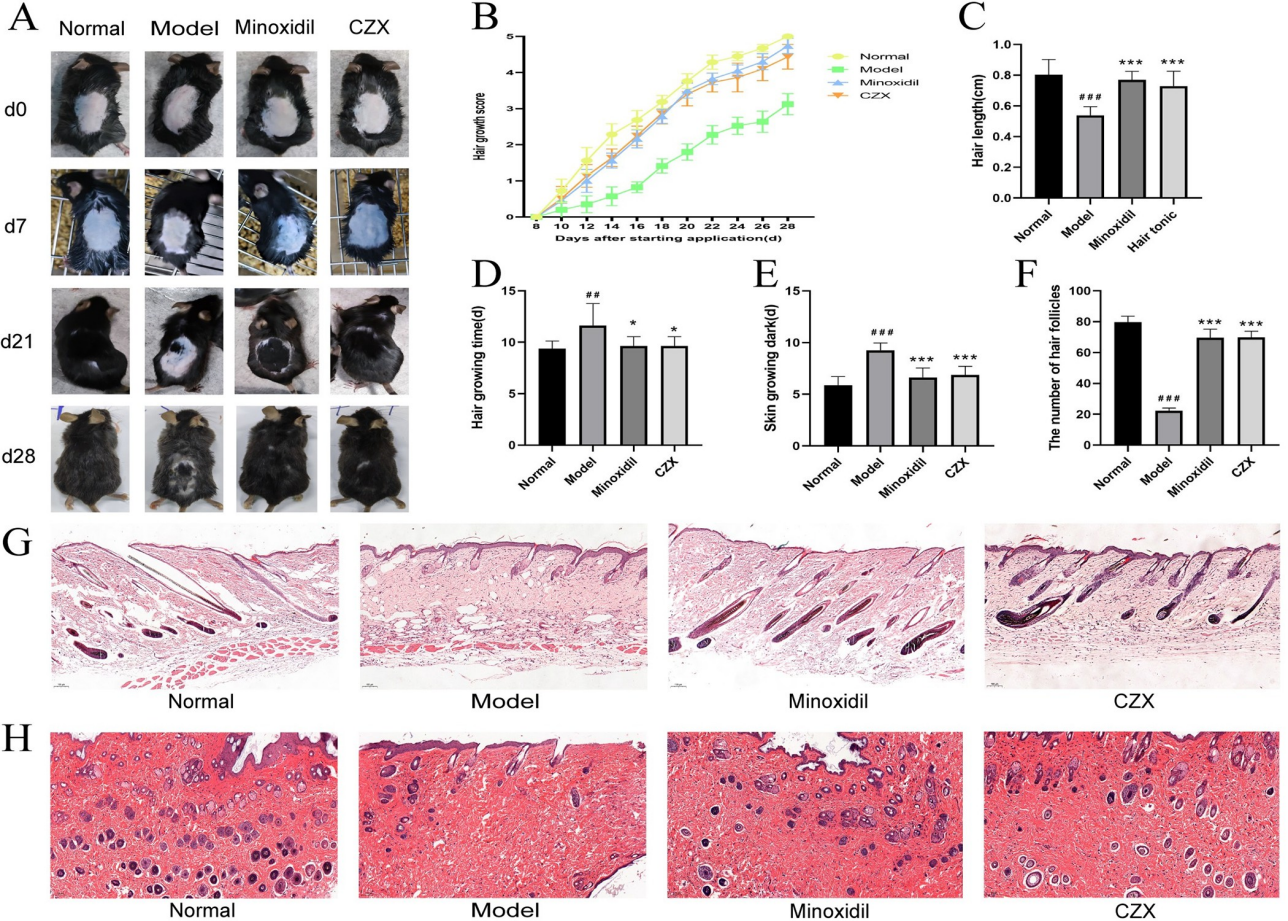
Effect of CZX on the hair cycle and hair follicle morphology in mice. (A) The backs of the mice were shaved and then treated with 95% ethanol, minoxidil and CZX. (B) Hair regrowth scores were determined every other day starting on the 8th day after treatment. (C) Average hair length of the mice in each group. (D) and (E) Skin regrowth times and skin darkening times. (F) The number of hair follicles. (G) and (H) Longitudinal and transverse sections of the dorsal skin. Digital images were captured using a 50× magnification light microscope. The results are presented as the mean ± SD. Compared with the normal group, ^##^*P <* 0.01, ^###^*P <* 0.001; compared with the model group, *P< 0.05, ***P < 0.001, n = 8.

After the dorsal hair of seven-week-old C57BL/6 mice was depilated, the skin of the mice in the telogen phase at this time was pink in colour. The skin turned black when it entered the anagen phase, and finally, the hair grew back (Fig 1A). As shown in Fig 1D and 1E, skin darkening and hair regrowth took significantly longer in the model group than in the normal group, suggesting that testosterone administration may delay hair regrowth. Moreover, the application of CZX significantly reduced the time required for the dorsal skin to darken and hair to regrow compared with the model group (*P <* 0.05, *P <* 0.001).

To further test the hair growth-promoting activity of CZX, hair growth was scored every other day from the 8th day after drug administration. It was found that the score of the CZX group was higher than that of the model group (Fig 1B). After the experiment, the average hair length of the mice was longer in the CZX group than in the model group (*P <* 0.001) (Fig 1C). The above results revealed that CZX showed significant hair growth promoting activity in an AGA mouse model.

### Effects of CZX on the histopathology of skin sections

The hair follicle morphology in the longitudinal dorsal skin sections is shown in Fig 1G. Mice in the normal, CZX and minoxidil groups had more hair follicles, mainly in the late anagen and early catagen stages, with significant melanin formation and downward growth into the deep dermis and subcutaneous tissues. The hair follicles of the model mice were sparse and mainly in the middle and late stages of catagen, with less melanin, located in the epidermis and superficial dermis. Hair follicles were counted using transverse sections (Fig 1H). The mean number of hair follicles in the model group was significantly lower than those in the normal, minoxidil and CZX groups (*P <* 0.001) (Fig 1F).

### Components of CZX

**Fig 2:**
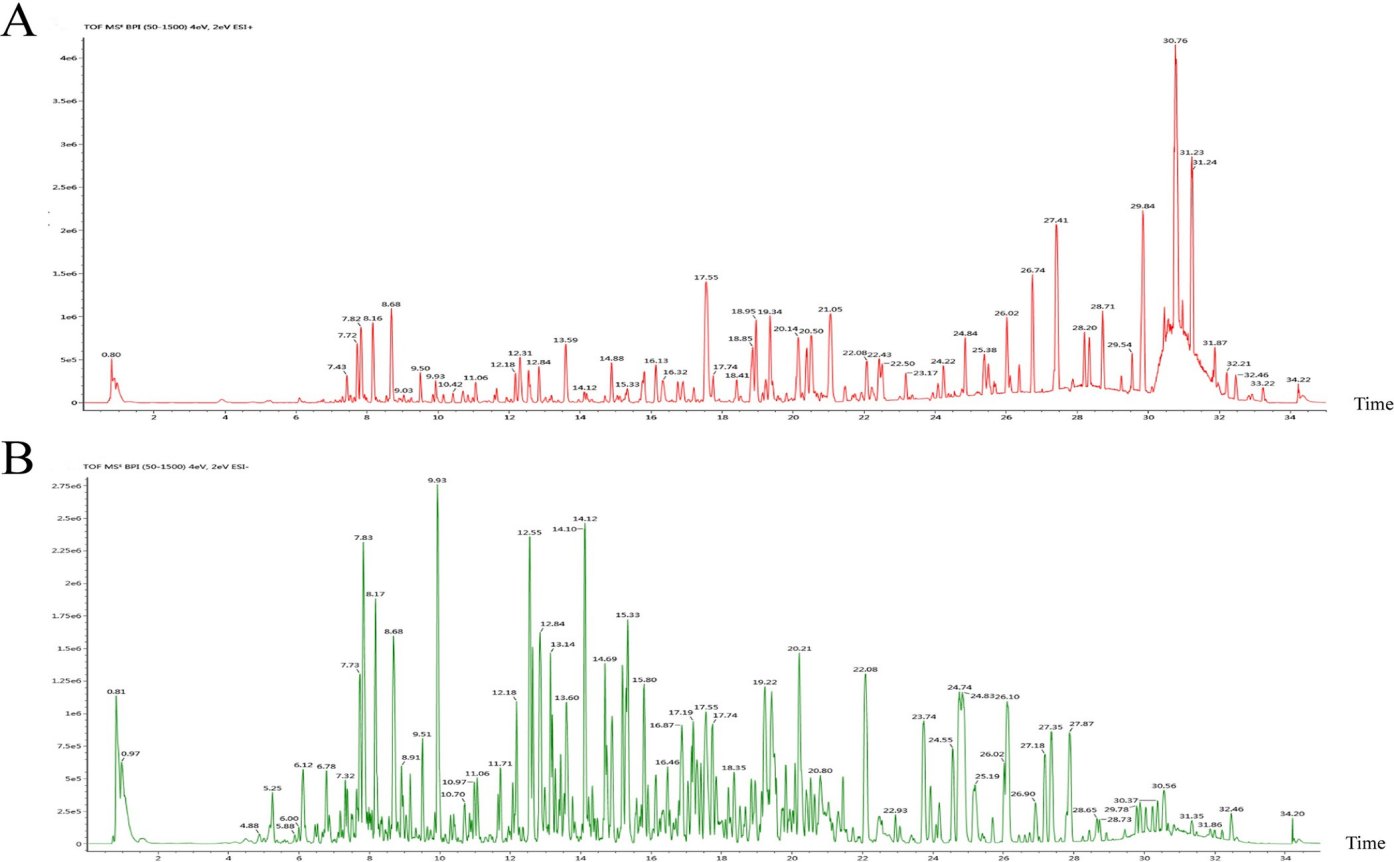
UPLC–Q–TOF/MS analysis of CZX. Total ion current base peak intensity (BPI) chromatogram in (A) positive mode and (B) negative mode.

Following UPLC–Q–TOF/MS analysis (Fig 2A and 2B), 69 chemical constituents were analysed with a self-built database and by literature comparison, and 35 active components were screened according to Lipinski’s rule of five [18] (Table 2), including 9 from Heshouwu, 12 from Huangqi, 10 from Danggui, 1 from Heizhima, 5 from Shengjiang, and 5 from Cebaiye. Among these, there were 3 common components between Danggui and Shengjiang; 1 common component between Danggui and Huangqi; 1 common component between Cebaiye and Huangqi; and 1 common component among Heshouwu, Cebaiye and Huangqi.

**Table 2:** Identification of the chemical constituents of CZX.

No.	Compound	Molecular formula	RT (min)	Measured Mass(m/z)	Error(mDa)	Adduct ions	Medicinal materials of possible origin
1	3-Hydroxy-9,10-dimethoxyptercarpan	C_17_H_16_O_5_	6.78	301.1078	0.7	+H	Huangqi
2	(+)-delta-Cadinene	C_15_H_24_	25.19	205.1952	0.2	+H, +Na	Shengjiang
3	Physcion	C_16_H_12_O_5_	12.21	285.0755	-0.2	+H	Heshouwu
4	Resveratrol	C_14_H_12_O_3_	10.85	229.0863	0.3	+H	Heshouwu
5	(E)-5-(4-Methoxystyryl)benzene-1,3-diol	C_15_H_14_O_3_	16.56	243.1014	-0.2	+H	Heshouwu
6	cis-3-Hexenyl benzoate	C_13_H_16_O_2_	21.05	205.1226	0.3	+H	Cebaiye
7	4-Ethylresorcinol	C_8_H_10_O_2_	21.05	139.0754	0.1	+H	Danggui
8	3’-Hydroxy-8-O-methylretusin	C_17_H_14_O_6_	15.64	315.0864	0.1	+H	Huangqi
9	Ligustilide	C_12_H_14_O_2_	22.07	191.1069	0.2	+H, +Na	Danggui
10	Emodin	C_15_H_10_O_5_	8.81	271.0604	0.3	+H	Heshouwu
11	4-Hydroxybenzaldehyde	C_7_H_6_O_2_	5.17	123.044	-0.1	+H	Shengjiang
12	Moupinamide	C_18_H_19_NO_4_	10.15	314.1387	0	+H	Heshouwu
13	Hyflavin	C_17_H_20_N_4_O_6_	6.28	377.1462	0.6	+H	Huangqi
14	Quercetin	C_15_H_10_O_7_	8.69	303.0505	0.5	+H	Heshouwu Cebaiye Huangqi
15	3-O-Methylquercetin	C_16_H_12_O_7_	8.78	317.066	0.4	+H	Huangqi
16	Isoferulic acid	C_10_H_10_O_4_	30.05	195.0654	0.3	+H	Huangqi Danggui
17	Phthalic anhydride	C_8_H_4_O_3_	32.36	149.0232	-0.1	+H	Danggui
18	Calycosin	C_16_H_12_O_5_	15.12	285.0758	0	+H	Huangqi
19	Tricin	C_17_H_14_O_7_	12.23	331.081	-0.2	+H	Heshouwu
20	Fallacinol	C_16_H_12_O_6_	12.27	301.0704	-0.2	+H	Heshouwu
21	Kaempferol	C_15_H_10_O_6_	9.5	287.0551	0.1	+H	Huangqi Cebaiye
22	Sedanolide	C_12_H_18_O_2_	17.42	195.1377	-0.2	+H, +Na	Danggui
23	Caryophyllene oxide	C_15_H_24_O	21.5	221.1897	-0.3	+H	Cebaiye
24	Betaine	C_5_H_11_NO_2_	0.94	118.0863	0	+H	Huangqi
25	Adenine	C_5_H_5_N_5_	0.92	136.0622	0.4	+H	Shengjiang Danggui
26	Coumarin	C_9_H_6_O_2_	22.93	147.0441	0	+H	Huangqi
27	Isoeugenol	C_10_H_12_O_2_	29.78	165.0909	-0.1	+H	Shengjiang Danggui
28	Isopimaric acid	C_20_H_30_O_2_	24.84	303.2315	-0.4	+H, +Na	Cebaiye
29	Isorhamnetin	C_16_H_12_O_7_	9.65	317.066	0.4	+H	Huangqi
30	Guaiacol	C_7_H_8_O_2_	12.31	125.0596	-0.1	+H	Danggui
31	Sesamol	C_7_H_6_O_3_	5.17	139.0391	0.1	+H	Heizhima
32	2’,4’-Dihydroxyacetophenone	C_8_H_8_O_3_	9.74	197.0456	0.1	+HCOO	Danggui
33	Formononetin	C_16_H_12_O_4_	15.64	313.0718	0	+HCOO	Huangqi
34	3-Methyl-1,8,9-anthracenetriol	C_15_H_12_O_3_	17.29	285.0776	0.7	+HCOO	Heshouwu
35	Vanillin	C_8_H_8_O_3_	8.93	197.0458	0.2	+HCOO	Shengjiang Danggui

### Key compounds from CZX and targets for treating AGA

**Fig 3:**
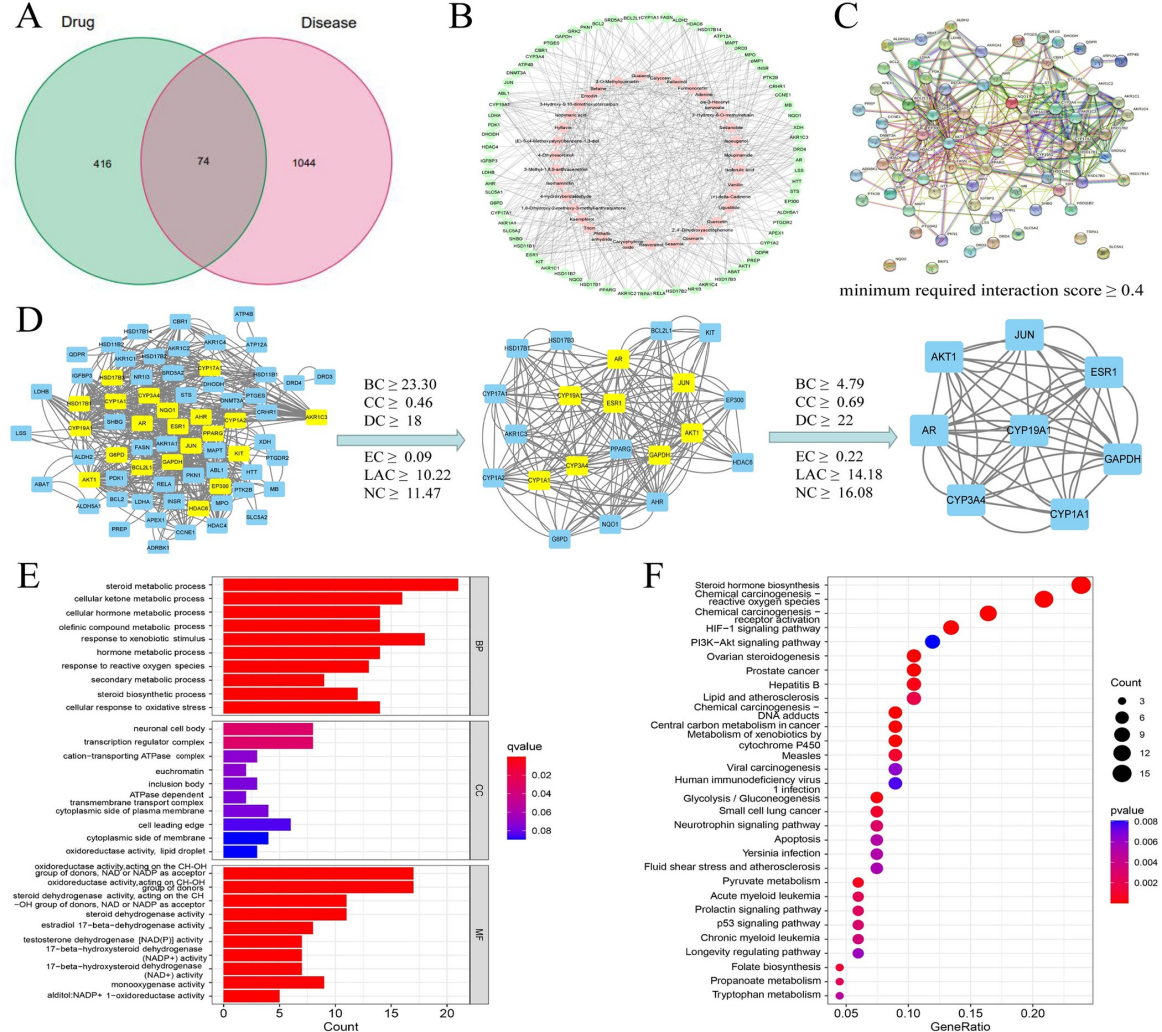
Network pharmacology predicted the key targets and potential signalling pathways of CZX for the treatment of AGA. (A) Venn diagram of the intersection targets between CZX and AGA. (B) Component-target network. (C) and (D) PPI analysis of the intersection targets. (E) GO enrichment analysis. (F) KEGG enrichment analysis.

The identified compounds from CZX were analysed by network pharmacology to predict the possible mechanisms of CZX to treat AGA. We identified 490 compound targets in total through the SwissTargetPrediction database based on the identified compounds of CZX (Table 2) and retrieved 1118 AGA-related targets from the GeneCards, OMIM, PharmGkb, and DrugBank databases. Finally, 74 intersection targets for CZX and AGA were obtained (Fig 3A).

Thirty-five active components and 74 intersection targets were imported into Cytoscape 3.8.0 software to construct the Component-target network, which consisted of 109 nodes and 459 edges (Fig 3B). The top 10 components of the degree value were resveratrol, quercetin, emodin, 4-hydroxybenzaldehyde, 4-ethylresorcinol, 2’,4’-dihydroxyacetophenone, tricin, Hyflavin, 3-O-methylquercetin and isoeugenol. It was speculated that these compounds may be the key components of CZX for the treatment of AGA.

Seventy-four intersection targets were uploaded to the STRING database (https://cn.string-db.org/) for PPI analysis. There were 74 nodes and 359 edges, of which 4 targets did not participate in protein interactions (Fig 3C). Cytoscape 3.8.0 software and the CytoNCA plug-in were used to analyse the topology of the intersection genes. The core genes screened were JUN, CYP1A1, CYP19A1, Akt1, CYP3A4, ESR1, AR, and GAPDH (Fig 3D). We speculated that these targets may be the key targets of CZX in AGA treatment.

### Prediction of CZX’s key effects on AGA

GO and KEGG enrichment analyses were conducted on 74 intersection targets. GO analysis consists of three aspects: BP, MF and cellular component (CC) (Fig 3E). According to the GO results, the mechanisms by which CZX can treat AGA were related to steroid metabolic process, cellular hormone metabolic process, transcription regulator complex, oxidoreductase activity and steroid dehydrogenase activity. The top 30 pathways enriched by KEGG are visually represented in a bubble plot (Fig 3F). The KEGG enrichment results indicated that the important pathways of CZX involved in the treatment of AGA included steroid hormone biosynthesis, the hypoxia-inducible factor-1 (HIF-1) signalling pathway, the phosphatidylinositol-3-kinase/protein kinase B (PI3K/Akt) signalling pathway, apoptosis, etc. Upon consultation of the relevant literature combined with enrichment analysis, we speculated that apoptosis and the PI3K/Akt signalling pathway may be important pathways by which CZX can treat AGA and verified this speculation through molecular experiments [19,20].

### Molecular docking results

**Fig 4:**
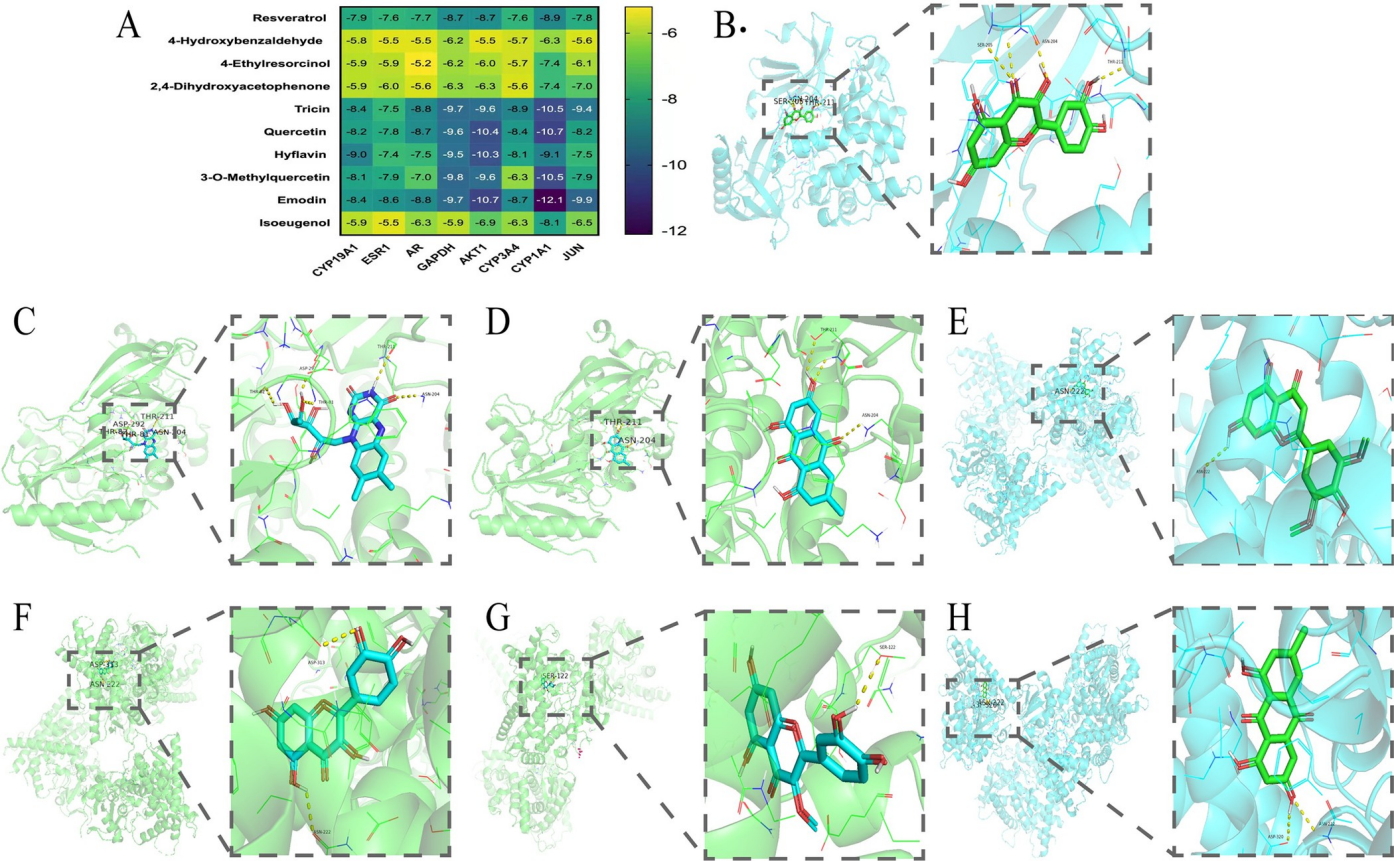
Molecular docking analysis. (A) Heatmap of the binding energies. (B) Quercetin-Akt1, (C) Hyflavin-Akt1, (D) emodin-Akt1, (E) tricin-CYP1A1, (F) quercetin-CYP1A1, (G) 3-O-methylquercetin-CYP1A1, and (H) emodin-CYP1A1.

The key targets (JUN, CYP1A1, CYP19A1, Akt1, CYP3A4, ESR1, AR, and GAPDH) obtained from PPI topological analysis were molecularly docked with the key components (resveratrol, quercetin, emodin, 4-hydroxybenzaldehyde, 4-ethylresorcinol, 2’,4’-dihydroxyacetophenone, tricin, Hyflavin, 3-O-methylquercetin and isoeugenol) in the Component-target network. Molecular docking analysis was performed using AutoDock Vina to obtain docking scores for proteins and components, and the results are represented as heatmaps. (Fig 4A). A lower docking score indicates a greater the affinity between the component and the protein. A docking score of under -5.0 kJ/mol indicates better binding [21]. As shown in Fig 4, Akt1 had high docking affinities with quercetin, Hyflavin, and emodin, with values of -10.4 kcal/mol, -10.3 kcal/mol and -10.7 kcal/mol, respectively. In addition, CYP1A1 had high docking affinities with tricin, quercetin, 3-O-methylquercetin and emodin, with values of -10.5 kcal/mol, -10.7 kcal/mol, -10.5 kcal/mol and -12.1 kcal/mol, respectively.

### Effects of CZX on the protein expression of key genes related to apoptosis and the PI3K/Akt pathway

**Fig 5:**
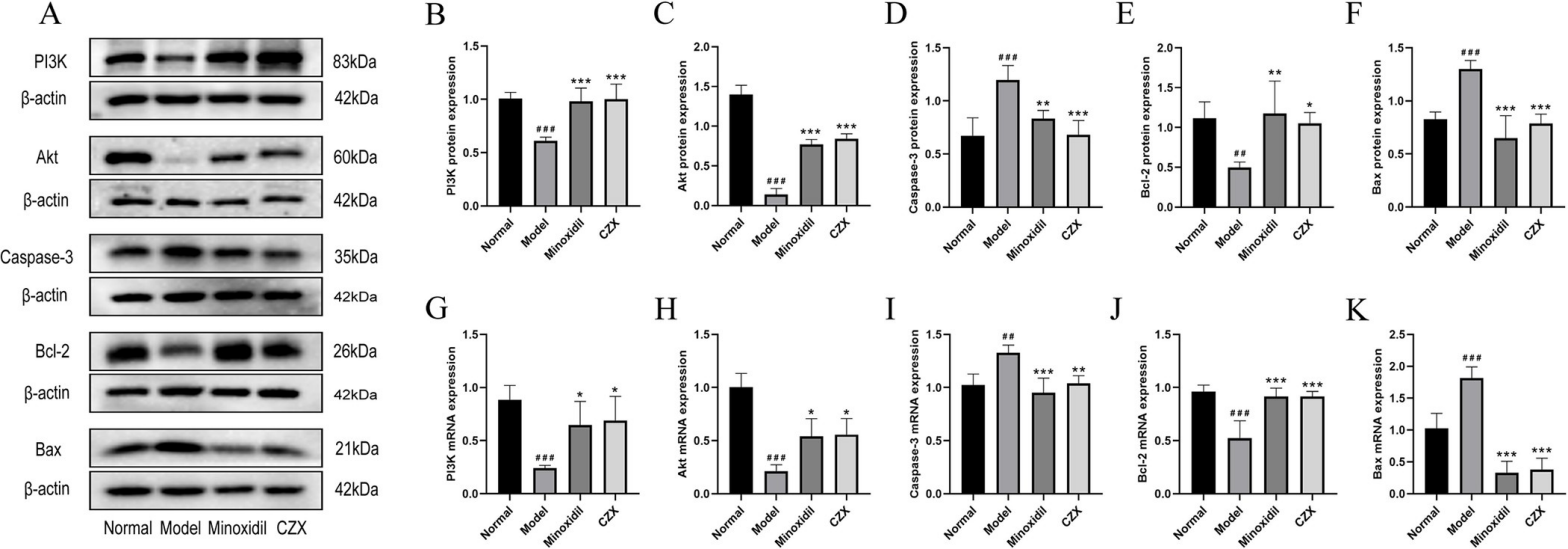
Effect of CZX on the protein and mRNA transcription levels of key genes related to apoptosis and the PI3K/Akt pathway. (A) Representative images of protein expression using Western blotting. Quantification of the protein expression levels of (B) PI3K, (C) Akt, (D) caspase-3, (E) Bcl-2 and (F) Bax from the Western blot analysis. mRNA expression levels of (G) PI3K, (H) Akt, (I) caspase-3, (J) Bcl-2 and (K) Bax from quantitative real-time PCR analysis. The results are presented as the mean ± SD. Compared with the normal group, ^##^*P <* 0.01, ^###^*P <* 0.001; compared with the model group, *P< 0.05, **P < 0.01, ***P < 0.001; n = 5.

To verify the reliability of the key signalling pathways (PI3K/Akt and apoptosis) predicted by network pharmacology, the expression of the proteins PI3K, Akt, Bcl-2, Bax, and caspase-3 in these two pathways was examined by Western blot analysis. As shown in Fig 5, the protein expression levels of PI3K, Akt and Bcl-2 in the skins of the mice from the minoxidil and CZX groups were significantly higher, and the protein expression levels of caspase-3 and Bax were significantly lower, than those in the model group (*P* < 0.05, *P* < 0.01, *P* < 0.001). According to the results, CZX could upregulate the protein levels of PI3K, Akt and Bcl-2 in mouse skin and downregulate the protein levels of caspase-3 and Bax.

### Effects of CZX on the mRNA transcription of key genes related to apoptosis and the PI3K/Akt pathway

Quantitative real-time PCR analysis was used to assess the mRNA expression of PI3K, Akt, Bcl-2, Bax and caspase-3. As shown in Fig 5, compared to the model mice, the mice in the minoxidil and CZX groups showed significantly higher levels of PI3K, Akt and Bcl-2 mRNA and significantly lower levels of caspase-3 and Bax mRNA in their skin (*P* < 0.05, *P* < 0.01, *P* < 0.001). These results indicated that CZX could activate PI3K, Akt and Bcl-2 transcript levels and inhibit caspase-3 and Bax transcript levels, which was consistent with the Western blot analysis.

## Discussion

AGA is a complex problem, and patients seeking medical treatment have limited options. Drug treatments such as minoxidil and finasteride often have side effects that make it difficult for patients to adhere to long-term treatment regimens. Receiving invasive treatments, such as platelet-rich plasma (PRP) injections and hair transplantation, is often difficult, usually need to be repeated and may lead to a costly investment [22–24]. Recently, topical herbal preparations have become more widely available because of their higher rates of compliance, fewer side effects, wider active spectrum and less expensive price [25,26]. As a topical TCM preparation, CZX is expected to be widely used in the complementary and alternative treatment of AGA in the future. For the purpose of verifying CZX’s therapeutic effects on AGA, we created an AGA mouse model by subcutaneously injecting testosterone propionate and then treating the mice with CZX. CZX effectively promoted hair growth in AGA model mice, which was verified by skin histological sections.

CZX contains many compounds, which makes it difficult to study its molecular mechanism. As a result, identifying its components is the first step. In accordance with the results of UPLC–Q–TOF/MS analysis, we identified resveratrol, 4-hydroxybenzaldehyde, 4-ethylresorcinol, quercetin, Hyflavin, isoeugenol, emodin and other compounds. Then, we used network pharmacological predictions combined with experimental verification to determine the main active components and molecular mechanisms of CZX for the treatment of AGA.

The network pharmacology results indicated that 10 compounds, including resveratrol, quercetin and emodin, might be the key components in CZX that allow it to exert therapeutic effects against AGA. Resveratrol is a natural polyphenolic compound with a variety of pharmacological and physiological activities, such as being an effective activator of antiaging genes to alleviate ageing-related functional decline in organisms. The activity of resveratrol to promote hair growth is considered a new antiaging effect [27,28]. Kubo C et al. found that resveratrol could promote hair growth by increasing the expression of IGF-1 and KGF in HaCaT cells and reducing the expression of TGF-β1 [29]. Moreover, a study has shown that resveratrol can stimulate hair matrix cell and dermal papilla cell (DPC) proliferation and protect hair follicle cells from environmental damage to promote hair follicle growth [30]. Therefore, resveratrol may promote hair growth through multiple pathways. It was reported that quercetin reduced inflammation-induced perifollicular damage in AGA by attenuating carbon monoxide (CO) production and also exerted anti-androgenic activity by inhibiting 5α-reductases and downregulating androgen receptors [31,32]. Kim J et al. found that quercetin can enhance cellular energy metabolism and increase secretion of growth factors to spike hair growth by activating the MAPK/CREB signaling pathway [33]. Therefore, quercetin-rich extracts exhibit promising hair growth-promoting activities [34]. Emodin has also been proven to slow down AGA’s progression, which may be due to its ability to inhibit 5α-reductase activity, reduce DHT levels in DPCs, and decrease the expression of two upregulated genes (TGF-β1, DKK-1) in AGA [35]. Because of the combination of these active compounds, CZX may have a better therapeutic effect on AGA.

The KEGG pathway enrichment results showed that CZX may play a therapeutic role in the treatment of AGA by regulating steroid hormone biosynthesis, apoptosis, the PI3K-Akt and HIF-1 signalling pathways, etc. One of the representative causes of hair loss is the failure to control apoptosis during catagen, leading to the continuous induction of apoptosis [36]. The most well-known apoptotic pathway is the intrinsic pathway mediated by mitochondria, which begins with Bax translocation to the mitochondrial membrane, which triggers the release of cytochrome c and prevents the release of Bcl-2. The released cytochrome c combines with procaspase 9 and other complexes to form apoptosomes. These apoptosomes then activate the caspase-3 signalling cascade, thereby inducing cell destruction [37]. Studies have shown that the PI3K/Akt pathway may play a key role in apoptosis. Activating PI3K/Akt can suppress apoptosis to treat related diseases [38–40]. Hair follicle regeneration is also impacted by the PI3K/Akt signalling pathway. Previous studies have found that when specific inhibitors are used to inhibit the PI3K/Akt pathway, hair follicle regeneration mediated by cultured epidermal stem cells (Epi-SCs) and skin-derived precursors (SKPs) is also significantly inhibited [41]. Therefore, activating the PI3K/Akt pathway to inhibit hair follicle cell apoptosis is a possible therapeutic strategy for AGA. In our study, CZX had a beneficial effect on hair growth by upregulating PI3K, Akt and Bcl-2 and downregulating Bax and caspase-3. Moreover, the results of this study are consistent with those obtained in previous studies. This suggests that CZX may promote hair growth by regulating the PI3K/Akt and apoptosis pathways.

In this study, UPLC–Q–TOF/MS was used to qualitatively analyse CZX, and the database provided by the instrument was then used to speculate the chemical composition of CZX. However, no chemical reference was used for comparison, and no atlas of the medicinal materials contained in CZX was tested. Therefore, we hope to detect the chemical composition of CZX through more accurate experiments. CZX is a complex TCM prescription that may play a role in treating AGA through multiple active components acting on multiple targets at the same time. This study verified only the PI3K/Akt and apoptosis pathways, and cannot fully represent the mechanism of CZX in the treatment of AGA. Therefore, further in-depth and comprehensive experiments are needed.

## Conclusion

In this study, we used UPLC–Q–TOF/MS to analyse the chemical components of CZX and combined it with network pharmacology to analyse the main components and mechanisms responsible for the effect of CZX on AGA. The findings reveal that CZX plays its therapeutic role mainly by regulating apoptosis and the PI3K/AKT pathway, which offers new approaches for treating and preventing AGA.

## Supporting information

S1 Raw images Boxes indicated parts used in the figure. (PDF)

S1 Graphical abstract (DOCX)
