# Supplementary material for: Caizhixuan hair tonic regulates both apoptosis and the PI3K/Akt pathway to treat androgenetic alopecia
Source: PLoS One. 2023 Feb 24;18(2):e0282427. doi: 10.1371/journal.pone.0282427 (PMC9956876; doi:10.1371/journal.pone.0282427)

Figure 5

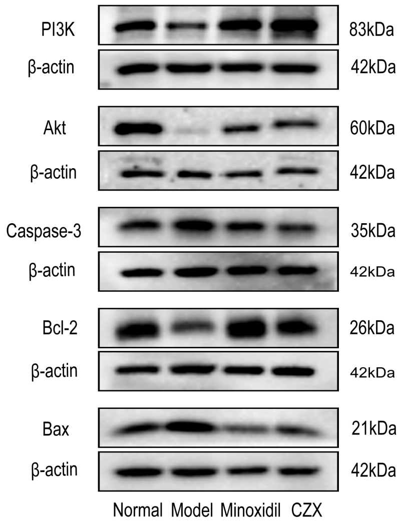

In western blot experiments, we used an ECL assay kit for exposure and a gel imager for capturing the image. In these pictures below, “A” represents the normal group, “B” represents the model group, “C” represents the minoxidil group and “D” represents the CZX group. Boxes indicated parts used in the figure 5.

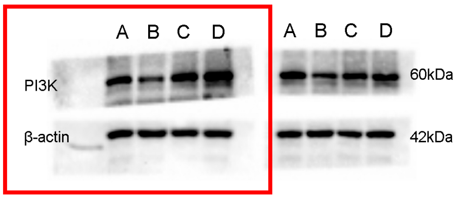

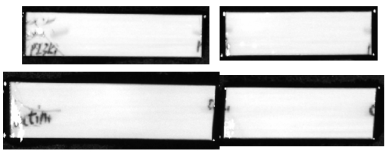

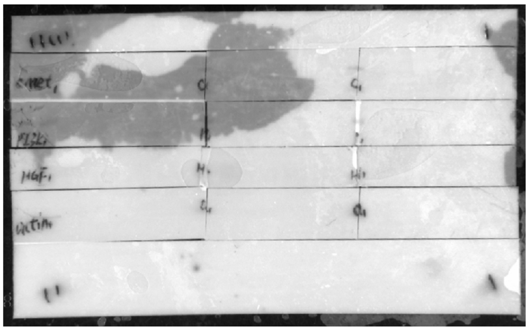

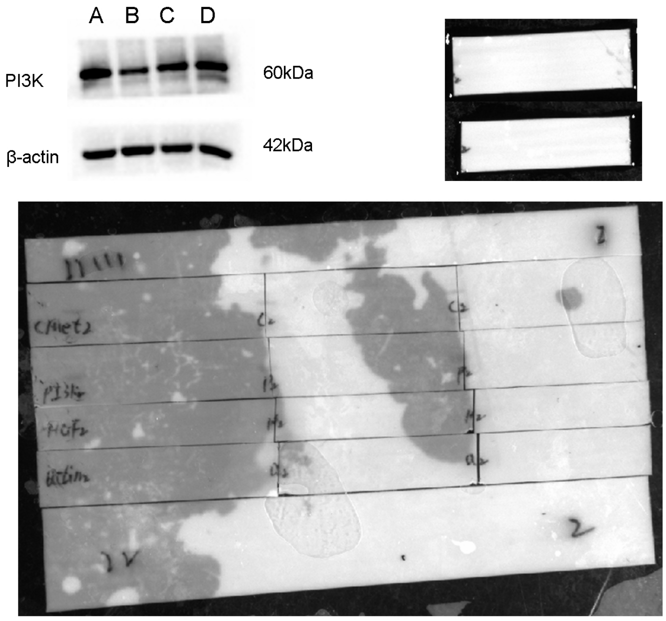
A B C D
PI3K
60kDa
β-actin
42kDa

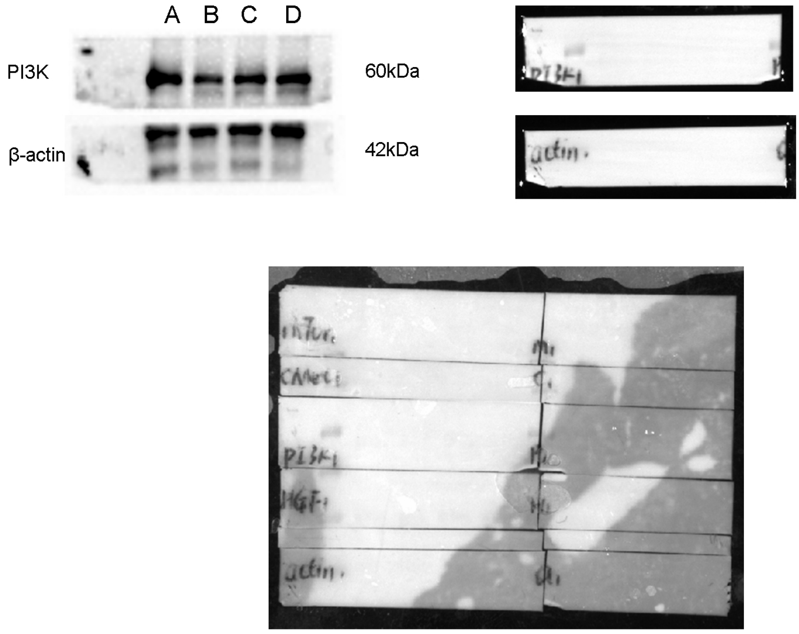
A B C D
PI3K
60kDa
β-actin
42kDa

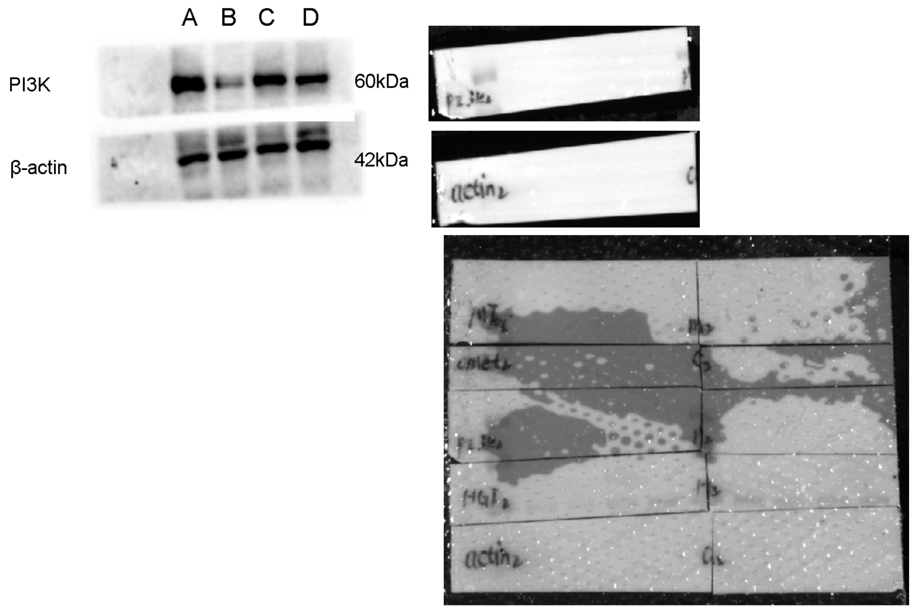
A B C D
PI3K
60kDa
β-actin
42kDa

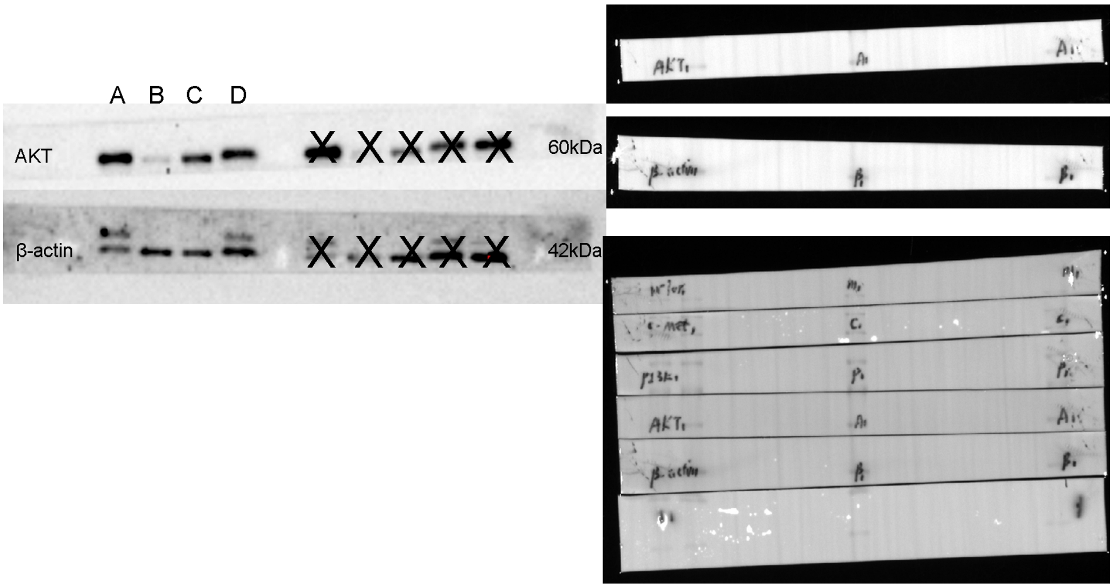
A B C D
AKT
60kDa
β-actin
42kDa

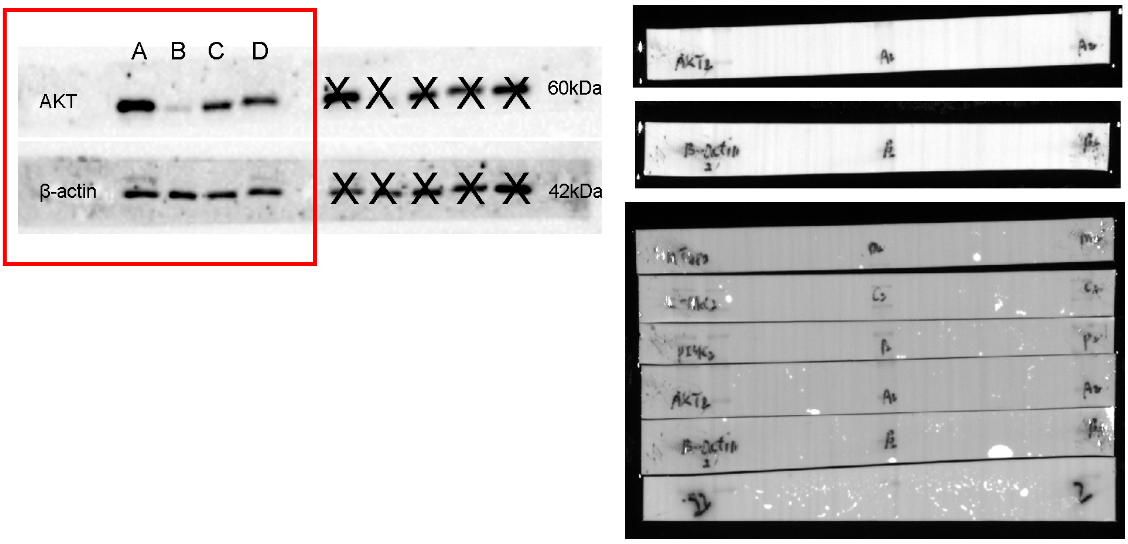
A B C D
AKT
60kDa
β-actin
42kDa

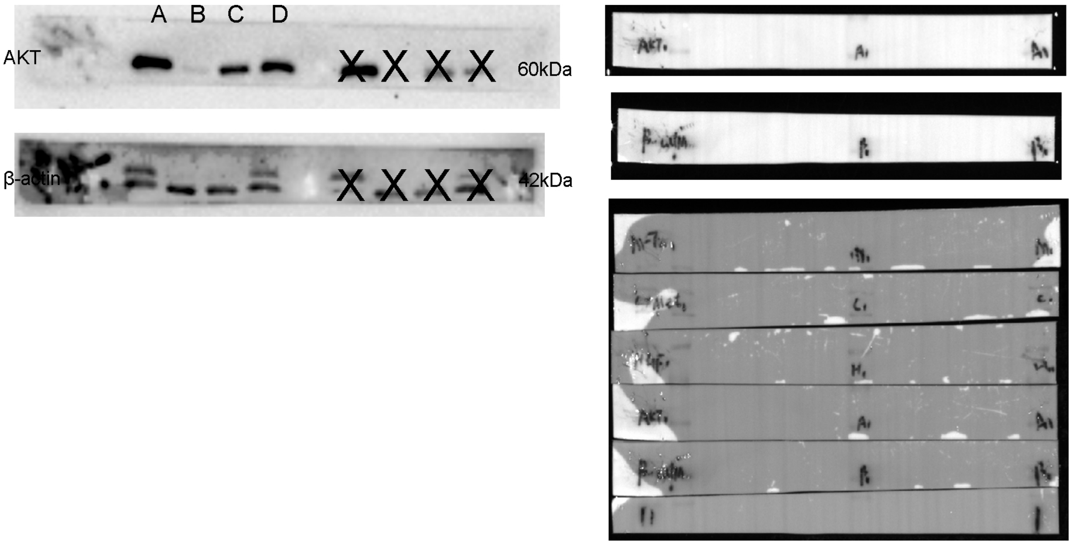
A B C D
AKT
60kDa
β-actin
42kDa

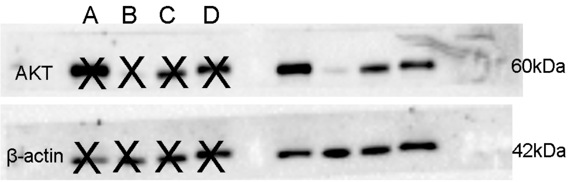
A B C D
AKT
60kDa
β-actin
42kDa

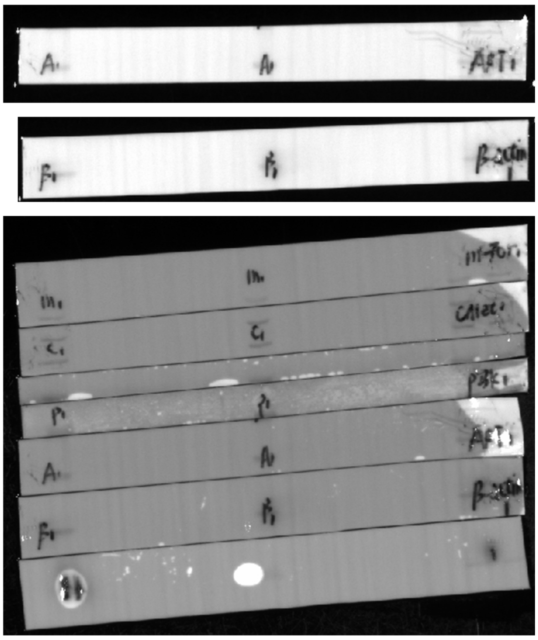

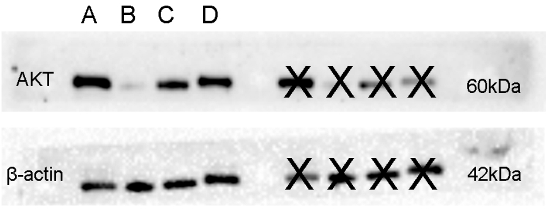
A B C D
AKT
60kDa
β-actin
42kDa

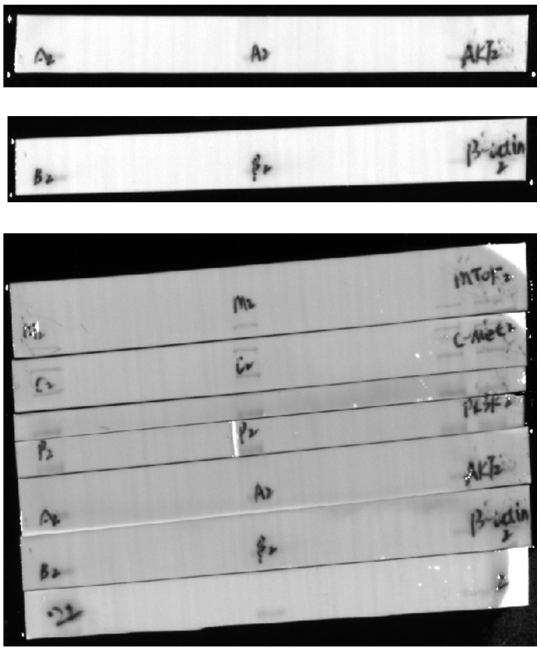

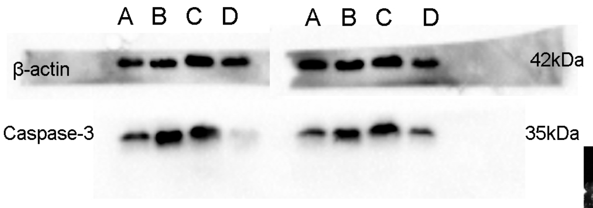
A B C D A B C D
β-actin
42kDa
Caspase-3
35kDa

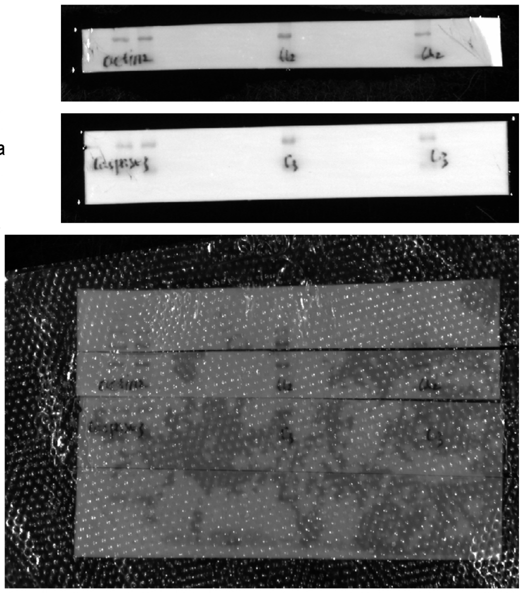

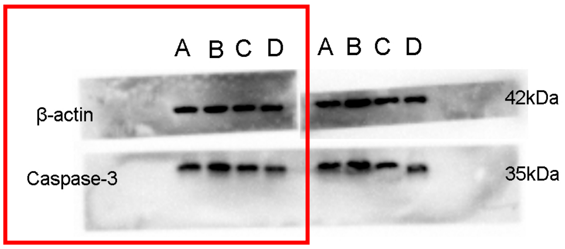
A B C D A B C D
β-actin
42kDa
Caspase-3
35kDa

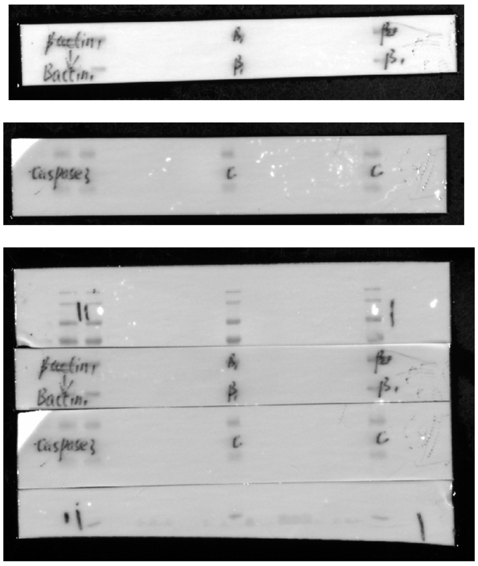

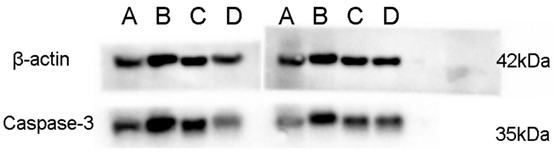
A B C D A B C D
β-actin
42kDa
Caspase-3
35kDa

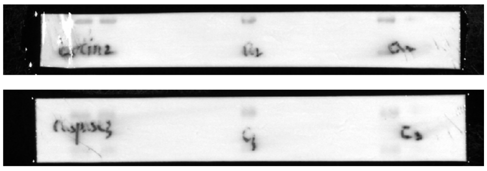

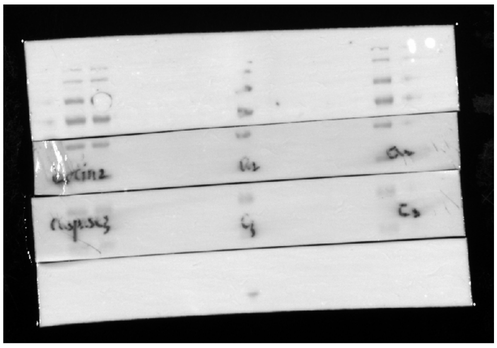

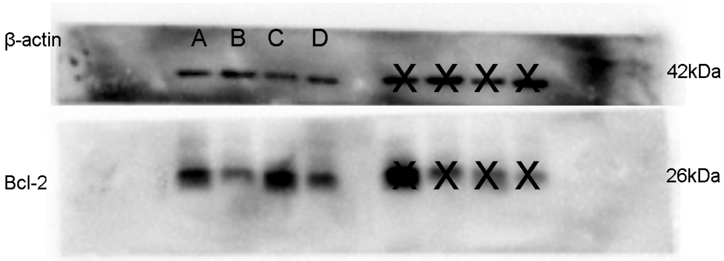
β-actin
A B C D
42kDa
Bcl-2
26kDa

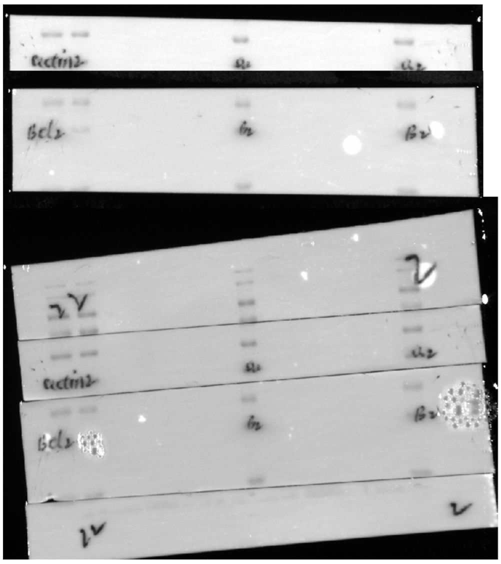

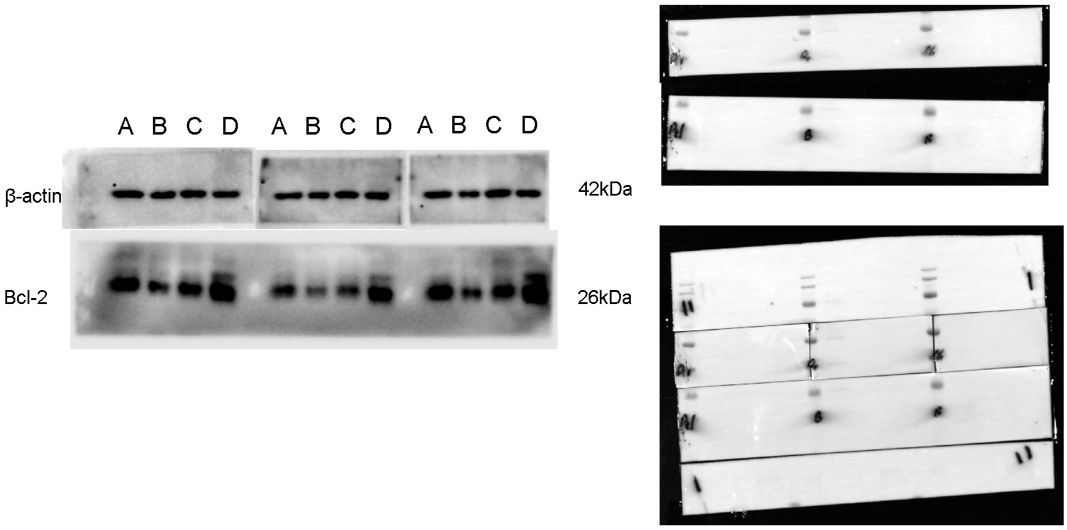
A B C D A B C D A B C D
β-actin
Bcl-2
42kDa
26kDa

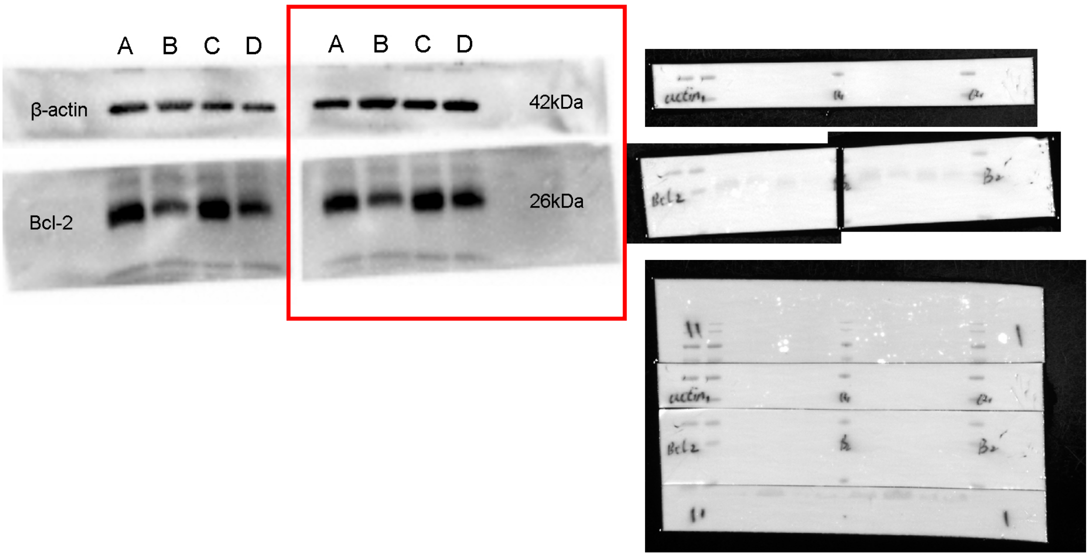
A B C D A B C D
β-actin
Bcl-2
42kDa
26kDa

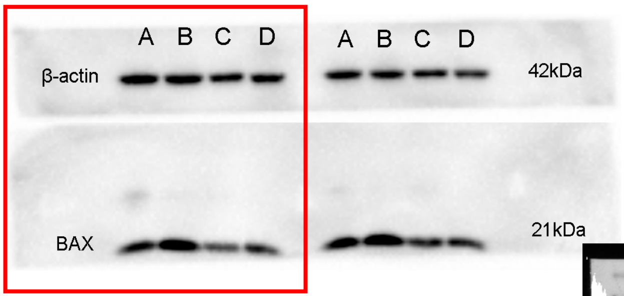
A B C D A B C D
β-actin
42kDa
BAX
21kDa

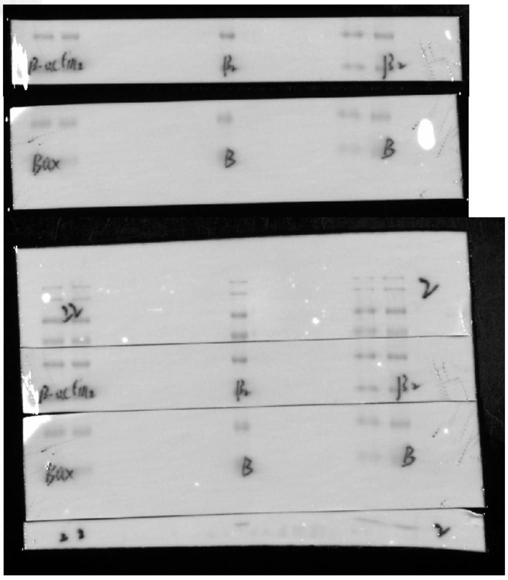

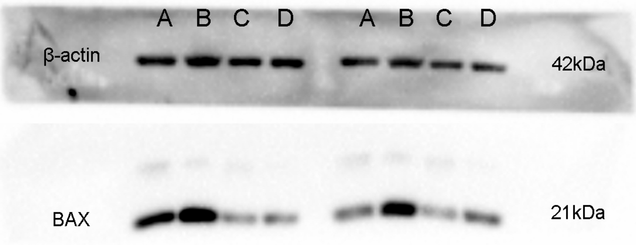
A B C D A B C D
β-actin
42kDa
BAX
21kDa

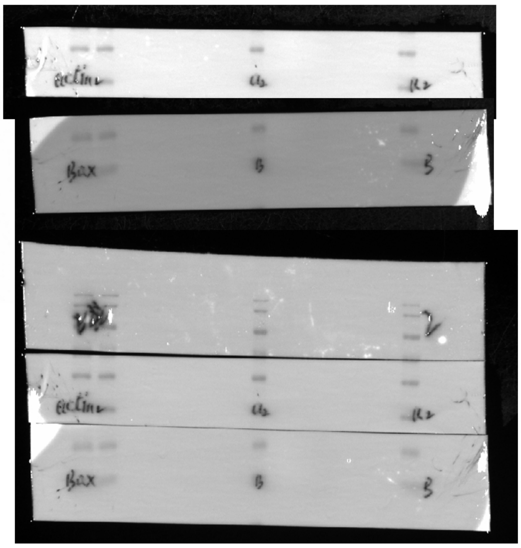

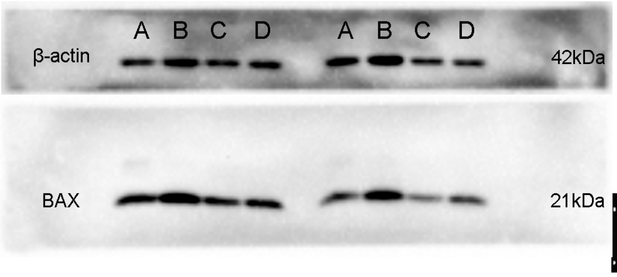
A B C D A B C D
β-actin
42kDa
BAX
21kDa

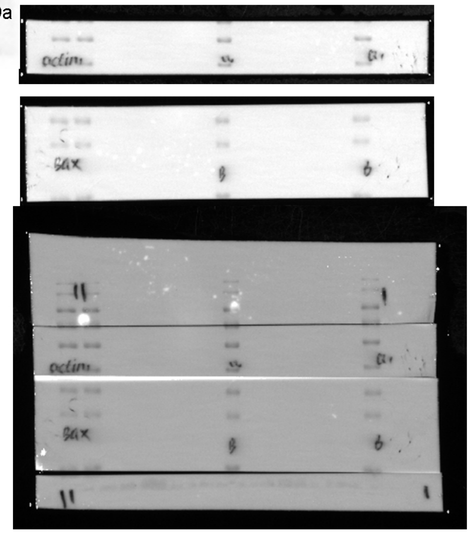

Supplement: S1 Raw images — (PDF) [file pone.0282427.s001.pdf]
