# Supplementary figures and images for: Caizhixuan hair tonic regulates both apoptosis and the PI3K/Akt pathway to treat androgenetic alopecia
Source: PLoS One. 2023 Feb 24;18(2):e0282427. doi: 10.1371/journal.pone.0282427 (PMC9956876; doi:10.1371/journal.pone.0282427)

### **Graphical abstract**


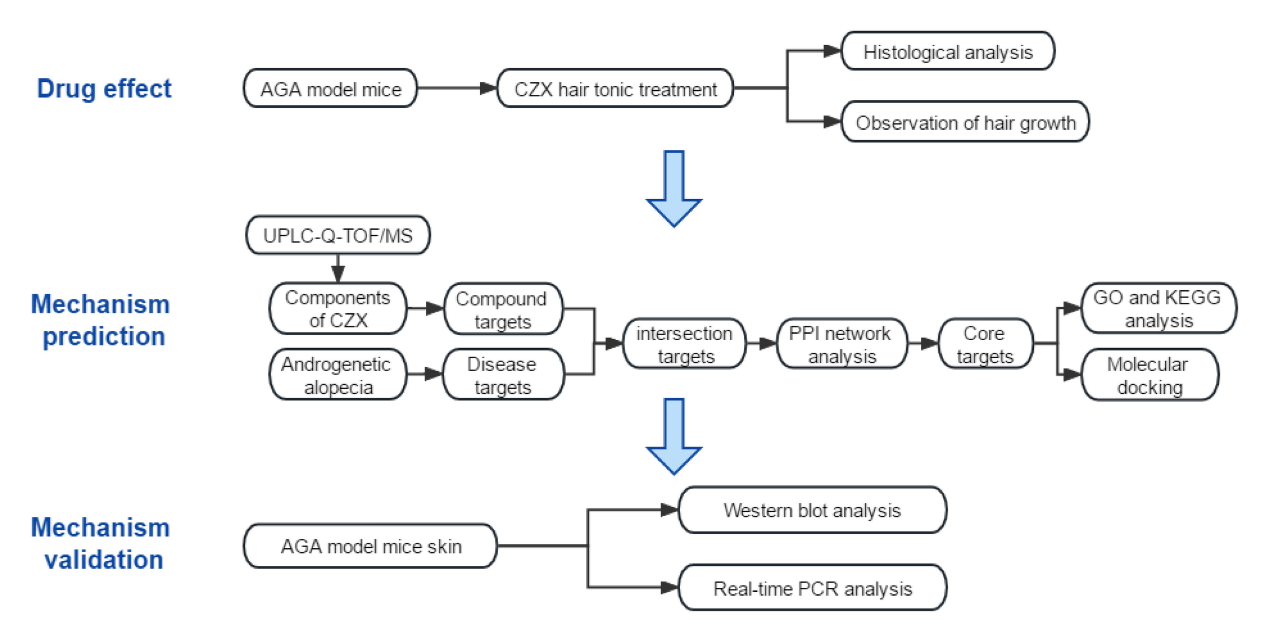

Supplement: S1 Graphical abstract — (DOCX) [file pone.0282427.s002.docx]
